# “Don’t know” scores should be considered when assessing violence risk for youth in acute institutions

**DOI:** 10.3389/fpsyt.2025.1705810

**Published:** 2026-02-11

**Authors:** Anniken Lucia Willumsen Laake, John Olav Roaldset, Tonje Lossius Husum, Stål Kapstø Bjørkly, Carina Chudiakow Gustavsen, Sara Teresia Grenabo, Øyvind Lockertsen

**Affiliations:** 1 Department of Nursing and Health Promotion, Faculty of Health Sciences, Oslo Metropolitan University, Oslo, Norway; 2 Centre for Research and Education in Forensic Psychiatry, South Eastern Norway Regional Health Authority, Oslo University Hospital, Oslo, Norway; 3 Faculty of Health Sciences and Social Care, Molde University College, Molde, Norway; 4 Department of Child and Adolescent Psychiatry, Oslo University Hospital, Oslo, Norway; 5 Youth Acute Child Welfare Institution, Oslo Municipality, Oslo, Norway

**Keywords:** violence risk screening, acute institutions, youth, “Don’t know” scores, violence

## Abstract

Commonly, relevant information to score violence risk assessment instruments is missing at the time of assessment. While there are indications that lack of information to score items, or “Don’t know” scores, has clinical relevance, these items are commonly omitted or treated as “No” scores in research. The Violence Risk Assessment Checklist for Youth (V-RISK-Y) is a screening instrument designed to identify violence risk in youth aged 12–18. The aim of this study is to assess whether “Don’t know” scores on V-RISK-Y are associated with increased risk for registered violent events for youth during acute institutional stays as compared to “No” scores. This study utilized data from the V-RISK-Y multicenter study, consisting of a sample of 517 youth from child and adolescent psychiatry and residential youth care institutions. The following secondary analyses were performed: (i) the frequencies of “Don’t know” and “No” scores for each item and registered violent events, and (ii) effect sizes from item-level logistic regression analyses of “Don’t know” scores with “No” scores as reference, controlling for sex and type of institution. Findings show more registered violent events for youth during institutional stay when items were scored as “Don’t know” as compared to “No”. Nine of the 12 items had odds ratios (ORs) above 1 for “Don’t know” for recorded violence when controlled for sex and type of institution. One item, “Lack of empathy”, reached significance. The positive ORs indicate that “Don’t know” scores may have clinical relevance despite not reaching statistical significance and that these scores should be considered both in clinical practice and in research.

## 1 Introduction

Rapid identification of violence risk is often required in acute institutional settings. For instance, in inpatient psychiatric units, a high proportion of violent episodes happen early during the institutional stay (1, 2). Furthermore, in residential care institutions for children and adolescents, youth may be placed in institutions involuntarily due to behavioral issues, which may signify violence risk (3). However, information relevant to determine risk may be unknown at admission point (4, 5). For example, in emergency departments, patient history might be unavailable at intake, and rapid collection of relevant information might be logistically challenging (5). While research assessing the relationship between “Don’t know” scores and violence risk is scarce, Eriksen and colleagues (6) found that “Don’t know” scores were significantly associated with violence in an inpatient psychiatric adult population. These findings were supported in a more recent archival study on V-RISK-10 (7). In research, items with “Don’t know” scores have typically been omitted or treated as missing in statistical analyses (6, 7), and missing data have commonly been excluded (e.g. 8, 9). However, risk assessment scores should be based on all instrument items, and omitting these items can alter the structure of the instrument and impact predictive accuracy (10). Furthermore, it is pointed out that whether lack of information to score items in violence risk assessments impacts the instruments’ performance is currently unknown (11). Thus, “Don’t know” scores may have implications for both clinical practice and research, but there is a scarcity of literature exploring this topic.

### 1.1 Approaches to violence risk assessment

Currently, there are two main approaches to structured violence risk assessment. First, actuarial risk assessments are conducted by rating the presence of empirically derived risk factors and calculating a score based on these factors (12). The risk assessment is determined solely based on this score, without including subjective evaluations (13). Second, the structured professional judgment (SPJ) approach also relies on validated risk factors, but incorporates clinical judgment (14). Violence risk is typically assessed as “Low”, “Moderate”, or “High” based on present risk factors as well as consideration of other relevant factors like setting and available resources (15). By allowing some professional discretion, SPJ tools allow for consideration of individual and dynamic factors in the risk assessment (16, 17). On the other hand, allowing for this flexibility may introduce bias and increase variability (18). Both types of instruments perform similarly in terms of predictive validity (15, 19). However, in treatment settings, the SPJ approach is typically preferred because the inclusion of individual factors can help inform treatment plans and violence risk management strategies (20, 21). Several violence risk assessments are used with youth populations (22). Most research in this field is conducted in forensic settings, but it is increasingly expected or mandated that identification of violence risk takes place in healthcare and social services (23).

### 1.2 Violence risk screening in acute institutional settings

While several violence risk assessments have been validated for use in mental health patient populations, they typically require training and lengthy administration (e.g. 24, 25). In acute institutions, however, time and resources to administer comprehensive risk assessments may not be available (26). Accommodating these settings, briefer violence risk screening instruments have been developed for adult populations to rapidly identify violence risk and implement relevant interventions if needed (27, 28). V-RISK-10 (29) is currently the internationally recommended violence risk screener for acute mental healthcare contexts (27). For children and adolescents, comparable screening tools for use in acute settings are largely absent. Therefore, the Violence Risk Assessment Checklist for Youth (V-RISK-Y) was developed.

V-RISK-Y is a 12-item violence risk screening instrument developed to identify violence risk in youth aged 12–18 in acute institutions (English version freely available from sifer.no). The interrater reliability of V-RISK-Y was assessed in a case vignette study, which found high agreement between raters for the overall risk assessment (30). The predictive validity of V-RISK-Y was assessed in two studies from a multicenter project in four child and adolescent psychiatric units and four residential youth care institutions. V-RISK-Y had good predictive accuracy for violent behavior during institutional stay (31), with eight individual items as significant predictors of violence (32). In these studies, “Don’t know” scores were weighted in analyses, and item scores coded as “No” = 0; “Don’t know” = 1; “Maybe/Moderate” = 2; and “Yes” = 3. Items should be scored with “Don’t know” in instances where raters do not have sufficient information to conclude on the presence of a risk factor. Thus, items with these scores signify unavailable information but are included in the risk screening. In contrast, missing data stems from unscored items. Linearity of the item variables was established using fractional polynomials. “Don’t know” scores were highly prevalent for several items, and logistic regression analyses indicated that “Don’t know” scorings had substantial effect sizes and significantly predicted violent events for three V-RISK-Y items (Poor insight, Lack of empathy, and Unrealistic planning) (32). In these analyses, however, sex and type of institution were not controlled for. Both were significant predictors of violent events in previous V-RISK-Y studies (26, 31).

### 1.3 Aim

The objective of this brief report is to explore the impact of “Don’t know” scores on the predictive accuracy of V-RISK-Y by expanding on findings from the V-RISK-Y multicenter project. Specifically, the aim is to compare the predictive validity of “Don’t know” scores (i.e., not enough information to score an item) and “No” scores (i.e., risk factor excluded). It is hypothesized that “Don’t know” scores will be more predictive of violence risk than “No” scores.

## 2 Methods

### 2.1 Design and participants

This study leveraged data from the V-RISK-Y multicenter study (see 31 for detailed study and sample description). The multicenter study had a naturalistic, prospective, observational design and included all youth (*n* = 517) with institutional stays in four acute child and adolescent inpatient units (*n* = 355) and four residential youth care institutions (*n* = 162). The sample consisted of 362 girls and 153 boys (missing *n* = 2), and the mean age was 15.2 years. The duration of data collection was planned for 1 year at each institution, but varied from 12 to 14 months due to logistical challenges caused by the COVID pandemic. While some participants had several institutional stays during the data collection period, only one stay for each youth is included in the study to avoid one person from counting several times in analyses.

### 2.2 Measures

Data included V-RISK-Y scorings at intake (baseline measure) and registered violent episodes during institutional stay (outcome measure). V-RISK-Y was administered by institutional staff upon admission, and scored interdisciplinary by at least two staff members if possible. Episodes of violence or threats during institutional stay were registered in a separate Violence and Threats (VT) form by staff present during the incident. Violence and/or threats were registered for 59 youth (27 girls and 32 boys). Violence was defined as “attacks against another person with the intent of causing harm” and included verbal and physical threats.

### 2.3 Ethical approval

Ethical approval for the study was granted by the Regional Committee for Medical and Health Research Ethics (REK ID: 218444) and by the Data Protection Officer at Oslo University Hospital (ID 20/01146). The approval granted exemption from obtaining informed consent from the youth or their guardians to participate in the study. Upon admission, youth and guardians were informed about the project study and given the right to withdraw from the study. There was no further interaction with the study for the youth and/or their guardians.

### 2.4 Statistical analyses

Power analyses with 5% significance level and 80% power were conducted by a statistician for the multicenter study (31). Analyses were based on data from the V-RISK-Y pilot study (26) and suggested a minimum of 156 participants. All statistical analyses were conducted in SPSS versions 30 and 31.

Secondary analyses were performed on data from the multicenter study. “Moderate” and “Yes” scores were excluded from analyses, so that only “No” and “Don’t know” scores were compared (logistic regression analyses reporting on all scoring options are included in the study assessing predictive validity of V-RISK-Y items in 32). Frequencies of “Don’t know” and “No” scores and corresponding registered episodes of violence and/or threats were extracted from the dataset. Differences in frequency of registered violence were assessed with Chi square or Fisher’s exact test (groups with <5 participants).

Logistic regression analyses were conducted to compare “No” and “Don’t know” scores for each V-RISK-Y item controlled for sex and type of institution (i.e., inpatient psychiatric units and residential youth care institutions).

## 3 Results

### 3.1 Descriptives

Number of “Don’t know” scores for V-RISK-Y items ranged from 0 to 12 across ratings. Frequencies of “No” and “Don’t know” scorings and registered violent events for the full sample are displayed in **Table 1**. The frequency of “No” scores ranged from 15.1% for V10 Future stress to 61.3% for V7 Suspicion. For “Don’t know” scores, the lowest frequency was 15.5% for V7 Suspicion, and the highest was 43.3% for V11 Severe trauma. Differences in rates of registered events of violence and threats between “No” and “Don’t know” scores were significant for V6 Poor insight (χ² = 11.8; *p* ≤ 0.001), V8 Lack of empathy (χ² = 13.0; *p* ≤ 0.001), and V9 Unrealistic planning (χ² = 4.37; *p* = 0.036).

**Table 1:** Frequency of “No” and “Don’t know” scores for individual V-RISK-Y items and registered violent events.

Item	“No”		“Don’t know”		Chi square (df) ^1^	*p* ^2^
	*n* (%)	Registered violence, *n* (%)^3^	*n* (%)	Registered violence, *n* (%)^3^		
V1 Violence	265 (51.3)	13 (4.9)	106 (20.5)	4 (3.8)		0.787
V2 Threats	240 (46.4)	11 (4.6)	132 (25.5)	11 (8.3)	2.15 (1)	0.142
V3 Substance abuse	285 (55.1)	27 (9.5)	94 (18.2)	10 (10.6)	0.109 (1)	0.742
V4 Severe symptoms	96 (18.6)	4 (4.2)	102 (19.7)	11 (10.7)		0.107
V5 Behavior	136 (26.3)	8 (5.9)	101 (19.5)	7 (6.9)	0.107 (1)	0.743
V6 Poor insight	207 (40.0)	10 (4.8)	125 (24.2)	20 (16.0)	11.8 (1)	**<0.001**
V7 Suspicion	317 (61.3)	27 (8.5)	80 (15.5)	10 (12.5)	1.12 (1)	0.274
V8 Lack of empathy	278 (53.8)	18 (6.5)	161 (31.1)	28 (17.4)	13.0 (1)	**<0.001**
V9 Unrealistic planning	205 (39.7)	15 (7.3)	189 (36.6)	26 (13.8)	4.37 (1)	**0.036**
V10 Future stress	78 (15.1)	3 (3.8)	143 (27.7)	16 (11.2)		0.079
V11 Severe trauma	72 (13.9)	6 (8.3)	224 (43.3)	27 (12.1)	0.761 (1)	0.383
V12 Own perception of violence risk	216 (41.8)	9 (4.2)	165 (31.9)	12 (7.3)	1.73 (1)	0.188

^1^Differences in registered violence between “No” and “Don’t know” scores.

^2^*p*-value for Chi square (degrees of freedom) for groups with ≥5 members and Fisher’s exact test for groups with <5 members.

^3^Percentage of youth with registered violent event within scoring category.

Bold values indicate significant with p-value < .05.

### 3.2 Logistic regression analyses

**Table 2** displays results from logistic regression analyses comparing “No” and “Don’t know” scores, controlling for sex and type of institution. In these analyses, “Don’t know” score for V8 Lack of empathy (OR = 2.1; 95% CI: 1.07, 4.12; *p* = 0.031) significantly increased risk of registered episodes of violence. In addition to item V8, OR values were above 1 for “Don’t know” scores for item V2 Threats (OR = 1.73; 95% CI: 0.710, 4.19; *p* = 0.229), V4 Severe symptoms (OR = 1.22; 95% CI: 0.313, 4.75; *p* = 0.775), V6 Poor insight (OR = 2.07; 95% CI: 0.825, 5.18; *p* = 0.121), V7 Suspicion (OR = 1.05; 95% CI: 0.449, 2.45; *p* = 0.913), V9 Unrealistic planning (OR = 1.37; 95% CI: 0.655, 2.87; *p* = 0.402), V10 Future stress (OR = 1.92; 95% CI: 0.500, 7.34; *p* = 0.342), V11 Severe trauma (OR = 1.04; 95% CI: 0.385, 2.82; *p* = 0.936), and V12 Own perception (OR = 1.35; 95% CI: 0.514, 3.54; *p* = 0.544).

**Table 2:** Logistic regression analyses of “No” and “Don’t know” scores for V-RISK-Y items and registered violent episodes, controlled for sex (ref = girls) and type of institution (ref = residential youth care).

Item (No = Reference)	OR (95% CI)	*p*
V1 Violence (*n* = 371)		
Sex	2.97 (1.10, 8.03)	**0.032**
Institution	0.334 (0.123, 0.910)	**0.032**
“Don’t know”	0.655 (0.202, 2.12)	0.481
V2 Threats (*n* = 372)		
Sex	2.50 (1.01, 6.21)	**0.048**
Institution	0.358 (0.147, 0.873)	**0.024**
“Don’t know”	1.73 (0.710, 4.19)	0.229
V3 Substance abuse (*n* = 379)		
Sex	2.46 (1.22, 4.96)	**0.012**
Institution	0.432 (0.204, 0.916)	**0.028**
“Don’t know”	0.816 (0.356, 1.87)	0.630
V4 Severe symptoms (*n* = 198)		
Sex	3.21 (1.02, 10.1)	**0.046**
Institution	0.307 (0.071, 1.33)	0.114
“Don’t know”	1.22 (0.313, 4.75)	0.775
V5 Behavior (*n* = 237)		
Sex	3.46 (1.15, 10.3)	**0.026**
Institution	0.297 (0.088, 1.01)	0.051
“Don’t know”	0.736 (0.229, 2.36)	0.606
V6 Poor insight (*n* = 332)		
Sex	3.54 (1.59, 7.91)	**0.002**
Institution	0.322 (0.126, 0.822)	**0.018**
“Don’t know”	2.07 (0.825, 5.18)	0.121
V7 Suspicion (*n* = 397)		
Sex	2.35 (1.17, 4.74)	**0.017**
Institution	0.492 8.231, 1.05)	0.066
“Don’t know”	1.05 (0.449, 2.45)	0.913
V8 Lack of empathy (*n* = 439)		
Sex	3.30 (1.71, 6.35)	**<0.001**
Institution	0.465 (0.237, 0.912)	**0.026**
“Don’t know”	2.10 (1.07, 4.12)	**0.031**
V9 Unrealistic planning (*n* = 394)		
Sex	3.65 (1.83, 7.27)	**<0.001**
Institution	0.431 (0.208, 0.897)	**0.024**
“Don’t know”	1.37 (0.655, 2.87)	0.402
V10 Future stress (*n* = 221)		
Sex	3.14 (1.16, 8.53)	**0.025**
Institution	0.272 (0.093, 0.796)	**0.018**
“Don’t know”	1.92 (0.500, 7.34)	0.342
V11 Severe trauma (*n* = 296)		
Sex	2.09 (0.974, 4.45)	0.058
Institution	0.279 (0.126, 0.618)	**0.002**
“Don’t know”	1.04 (0.385, 2.82)	0.936
V12 Own perception of violence risk (*n* = 381)		
Sex	1.48 (0.569, 3.83)	0.423
Institution	0.441 (0.168, 1.16)	0.097
“Don’t know”	1.35 (0.514, 3.54)	0.544

Bold values indicate significant with p-value < .05.

Sex was significant for all items except V11 Severe trauma and V12 Own perception. When type of institution was added to the model, OR was significant for all items except V4 Severe symptoms, V5 Behavior, V7 Suspicion, and V12 Own perception.

## 4 Discussion

### 4.1 “Don’t know” scores and registered violence

The proportion of youth registered with violent events during their institutional stay was higher for items scored “Don’t know” as compared to “No” for all items except V1 Violence (see **Table 1**). The only significant items were V6 Poor insight, V8 Lack of empathy, and V9 Unrealistic planning. Eight of the 12 items had more than 20% “Don’t know” scores, including the three significant items. Particularly high rates of “Don’t know” scores are seen for V11 Severe trauma (43.3%), V9 Unrealistic planning (36.6%), V12 Own perception (31.9%), and V8 Lack of empathy (31.1%). These findings emphasize that several items may be challenging to score conclusively as “No”, “Maybe/Moderate”, or “Yes” at intake, and that this lack of information may be related to increased likelihood of registered violent events. The lack of available information when scoring violence risk assessment instruments in clinical settings has been highlighted in previous literature (4, 5).

In research, attempts are often made to minimize the amount of unavailable data, whereas in clinical practice, this is not possible (11). Missing data increases uncertainty in predictions of violence risk (33). Notably, the three items that reached significance are dynamic factors, not historical. Thus, they require observation to determine and may be prone to subjectivity in scoring. For instance, lack of empathy is an intrapsychic phenomenon, which may be difficult for the rater to score based on the first impression of the youth. Consequently, this item may be more prone to a “Don’t know” score. It is possible these items are more challenging for some staff members to score conclusively and rapidly at intake. Simultaneously, given the statistical significance of the “Don’t know” score, these items might be particularly important to consider when assessing risk level and information to score the items is lacking.

### 4.2 Effect sizes may have clinical relevance

Findings from item-level logistic regression analyses show that, when controlled for sex and type of institution, 9 of the 12 items had odds ratios (ORs) exceeding 1 (see **Table 2**). Exceptions were items V1 Violence, V3 Substance abuse, and V5 Behavior. Most items have substantial effect sizes, meaning that the odds of a registered violent event is increased when an item is scored “Don’t know” as compared to “No”. In the item analyses conducted by Laake et al. (31), “Don’t know” scores significantly predicted registered violent events for three items. In the secondary analyses performed in the present study, where only “No” and “Don’t know” scores are compared and sex and type of institution are controlled for, only V8 Lack of empathy remained significant. The high ORs for boys show that the effect of sex explains some of the variance in registered violence. “Don’t know” scores may be of clinical relevance despite not reaching statistical significance (e.g. 34). Importantly, risk assessment items scored with “Don’t know” does not necessarily mean that the item is not present (i.e., a “No” score), but that the information needed to score the item conclusively is lacking at the time of instrument administration (6, 7).

### 4.3 Considering unavailable information in violence risk screening

Findings indicate that items scored as “Don’t know” could be interpreted as indicators of elevated uncertainty and considered as potentially contributing towards risk. While further research is needed, one potential implication of these findings is that they may inform decision support for clinicians conducting violence risk screenings. The nature of SPJ instruments, where Low–Moderate–High risk level is assessed based on item scores and evaluation of other relevant information, rather than a cutoff score, allows for individual evaluation of the relevance of “Don’t know” scores. When SPJ instruments are administered and data to score items are unavailable, clinicians must rely more heavily on subjective evaluation (35). On the other hand, actuarial tools typically have strong predictive validity, objectivity, and consistency (17). If considered in actuarial tools, “Don’t know” would contribute towards the cutoff score for violence risk. However, actuarial instruments have limited ability to capture dynamic and idiosyncratic factors relevant for risk management strategies (13).

### 4.4 Handling “Don’t know” scores and missing data in research

In addition to the clinical implications discussed above, findings may impact handling missing scores in statistical analyses. While transparency about missing data in violence risk assessment instruments is encouraged (36), handling of missing data is rarely reported (37). To determine whether weighting of missing items is appropriate, the relationship between missing data and “Don’t know” scores must be assessed further. While there are some differences between a missing score (not scored) and a “Don’t know” score (scored based on lack of information), they both signify that information about an item is unavailable. Commonly, studies sum up the available risk factors for risk assessments and dismiss missing items in analyses (10); however, Eriksen et al. (7) found it more accurate to include the “Don’t know” scores in analyses and weight them. Hopefully, findings from this study can further the understanding of how best to deal with missing data in research on violence risk assessment.

### 4.5 Limitations and future directions

Several limitations should be considered when interpreting findings from this study. First, because this was a naturalistic study, external variables were not controlled for. Specifically, the data collection overlapped with the pandemic, which may have interfered with generalizability due to COVID measures. Furthermore, it is possible that the implementation of V-RISK-Y led to heightened emphasis on violence risk and risk management, potentially preventing violent events in the participating institutions. Moreover, we do not have a way of accounting for subjectivity and staff dependency in violence risk screening evaluations and in registration of violence, and particularly in registration of threats. The generalizability of findings is further limited to the types of settings assessed. The number of observations in each group varies, and some groups in stratified analyses have small sample sizes. Additional limitations of the multicenter study are discussed in Laake et al. (31).

Despite study limitations, findings are a step towards mapping out the impact of “Don’t know” scores in violence risk assessment, which should fuel further exploration in additional clinical and research settings. In particular, the clinical implications of missing information should be mapped out.

## Data Availability

The data analyzed in this study is subject to the following licenses/restrictions: Restrictions due to confidentiality. The dataset is kept at an encrypted server. Requests to access these datasets should be directed to JR, johnolr@gmail.com.
